# Supplementary material for: Cooperative atomic motion during shear deformation in metallic glass
Source: Nat Commun. 2026 Jan 12;17:1604. doi: 10.1038/s41467-026-68308-4 (PMC12905200; doi:10.1038/s41467-026-68308-4)
Supplement: Supplementary file 1 — Supplementary Information [file 41467_2026_68308_MOESM1_ESM.pdf]

Supplementary Information: Cooperative atomic motion during shear deformation in metallic glass

Yoshinori Shiihara[1,*], Takuya Iwashita[2,*], Nozumu Adachi[3], Yoshikazu Todaka[3], Takeshi Egami[4,5,6,*]

[1] Graduate School of Engineering, Toyota Technological Institute, Nagoya, Aichi 468-8511, Japan
[2] Department of Science and Technology, Oita University, Oita 870-1192, Japan
[3] Department of Mechanical Engineering, Toyohashi University of Technology, Toyohashi, Aichi 441-8580, Japan
[4] Department of Materials Science and Engineering and Department of Physics and Astronomy, University of Tennessee, Knoxville, Tennessee 37996, USA
[5] Department of Materials Science and Engineering and Department of Physics and Astronomy, University of Tennessee, Knoxville, Tennessee 37996, USA
[6] Materials Science and Technology Division, Oak Ridge National Laboratory, Oak Ridge, Tennessee 37831, USA

*Correspondence: shiihara@toyota-ti.ac.jp, tiwashita@oita-u.ac.jp, egami@utk.edu

Supplementary Note 1. Frozen-atom analysis and artificial perturbations

Before the development of frozen-atom analysis, several studies introduced artificial manipulations to probe the physics of disordered structures. Pinning is one such approach: Berthier *et al.* constrained the motion of randomly selected atoms under NVT conditions and thereby revealed hidden static order (point-to-set correlations) in glass forming liquids[2]. Bhowmik *et al.* applied pinning during AQS deformation of a model amorphous solid, showing that frozen-atoms suppress avalanche-like plastic events[3]. Barbot *et al.* instead constrained the surroundings of a local region inside model amorphous solids and imposed affine deformation to forcibly trigger STZs, providing a map of local yielding propensity[1]. Xu *et al.* proposed to remove the contribution of specific pair bonds from the force constant matrix to identify those whose elimination directly triggers instabilities[5].

Frozen-atom analysis likewise employs artificial manipulation, but differs from pinning in that it does not suppress deformation indiscriminately throughout the AQS process. Compared with Barbot's method, the distinction lies in whether the constraint is applied inside or outside the STZ, while Xu's approach identifies critical bonds related to Local Configurational Excitation (LCE) rather than cooperative cores. The essential novelty of frozen-atom analysis is its ability to extract cooperative motion directly, a feature not accessible by previous methods. This indicates that frozen-atom analysis and these methods are complementary, and their combination may yield further insights into glass physics.

Supplementary Note 2. Preparation of the model glass structure

As described in the Methods section, in this study a velocity distribution corresponding to 2000 K was applied to the B2 structure of $Cu_{50}Zr_{50}$, and the system was directly cooled to 0 K at a rate of 10^9 K s^{-1} without an intermediate annealing stage. The transient data of the average total energy per atom and the average potential energy per atom obtained during this process

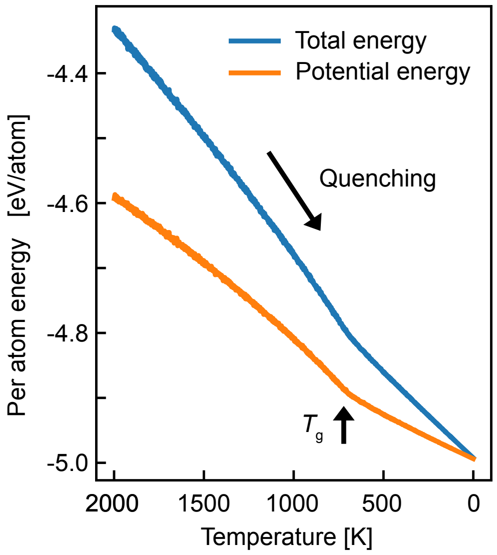

Supplementary Figure 1. Preparation of the model glass: evolution of per-atom total and potential energies during rapid quenching. The glass transition temperature, T_g, is estimated to be $\sim 750\,\mathrm{K}$ from the change in slope of the potential-energy curve.

are shown in Supplementary Figure 2. The potential energy exhibited a clear change in slope at around 750 K, from which the glass transition temperature T_g of this model glass was estimated to be approximately 750 K. Since the initial temperature of 2000 K is well above this value, the system can be regarded as having been quenched from a fully equilibrated liquid state. At 0 K, the total and potential energies coincide, indicating that the kinetic energy has vanished (less than 2 meV/atom). After cooling, the atomic structure and simulation cell were relaxed using the conjugate gradient method until all stress components converged to below 0.002 MPa. The final cell configuration of the $Cu_{50}Zr_{50}$ glass after quenching and relaxation is provided in Supplementary Table 1 to ensure reproducibility. The simulation box has an edge length of approximately 65.4 Å and is nearly cubic, but with small tilt factors, indicating a slightly triclinic geometry. The system density was 7.38 g/cm^3 after quenching and relaxation. The system density and T_g obtained here are consistent with those reported in Ref. [4] where the same Embedded-Atom Method (EAM) potential was employed, showing no significant discrepancy.

Supplementary Table 1. Final cell parameters of the $Cu_{50}Zr_{50}$ glass after quenching and relaxation.

Vector	x (Å)	y (Å)	z (Å)
**a**	65.4106	0.0098	-0.0314
**b**	0.0060	65.2043	0.0473
**c**	0.0000	0.0000	65.2881

Supplementary Note 3. Scalability of frozen-atom analysis

In terms of big-O notation with the number of atoms N, conventional molecular dynamics simulations scale as $O(N)$, whereas performing frozen-atom analysis by freezing every atom scales as $O(N^2)$. To reduce the computational burden, we applied the frozen-atom analysis only to atoms that exhibited a displacement norm above a certain threshold (e.g., 0.01 Å) during the

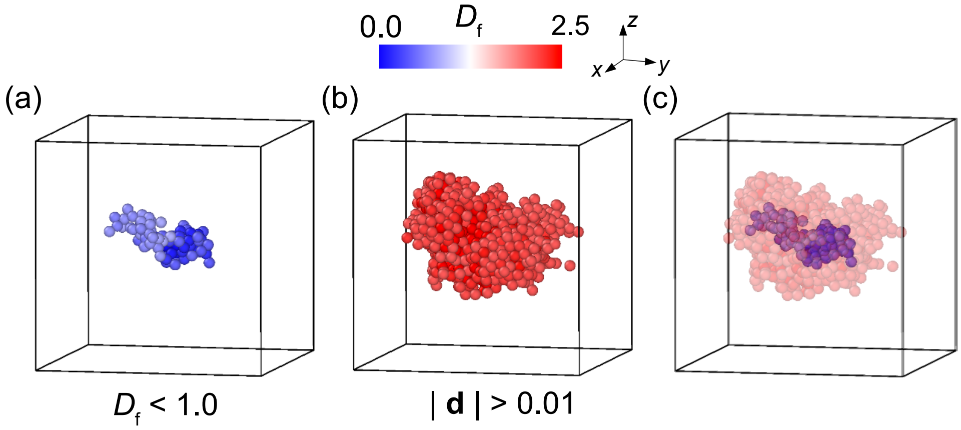

Supplementary Figure 2. Example of the computational cost reduction in frozen-atom analysis, illustrated for the second event under $+xy$ shear. (a) The STZ core (96 atoms) identified as the group of atoms with D_{f}. (b) The set of atoms that exhibited displacement norm $|\mathbf{d}| > 0.01$ (1204 atoms). (c) Superposition of (a) and (b). As seen in (c), the STZ core can be determined by applying the frozen-atom analysis only to the subset of atoms with relatively large displacements.

stress-drop event. Using atomic displacement as an initial screening step made it possible to greatly reduce the computational cost of the frozen-atom analysis. While an STZ core contains at most about 100 atoms, the threshold was chosen so that an atomic group roughly ten times larger was included for analysis. An illustrative example is shown in the figure below, where the STZ core is fully contained within the selected group. Test calculations for 20 events under $+xy$ and $-xy$ shear confirmed that the STZ cores identified by this efficient frozen-atom analysis are identical to those obtained by full analysis. Because the size of the STZ core is independent of the total number of atoms in the system, it is evident that freezing all atoms is unnecessary. With this refinement, the frozen-atom analysis can be regarded as an $O(N)$ algorithm, which makes the method scalable to large systems.

References

[1] A. Barbot, M. Lerbinger, A. Hernandez-Garcia, R. García-García, M. L. Falk, D. Vandembroucq, and S. Patinet. Local yield stress statistics in model amorphous solids. *Phys. Rev. E*, 97(3):033001, Mar. 2018.

[2] L. Berthier and W. Kob. Static point-to-set correlations in glass-forming liquids. *Phys. Rev. E*, 85(1):011102, Jan. 2012.

[3] B. P. Bhowmik, P. Chaudhuri, and S. Karmakar. Effect of Pinning on the Yielding Transition of Amorphous Solids. *Phys. Rev. Lett.*, 123(18):185501, Oct. 2019.

[4] P. Wen, B. Demaske, D. E. Spearot, S. R. Phillpot, and G. Tao. Effect of the initial temperature on the shock response of Cu50Zr50 bulk metallic glass by molecular dynamics simulation. *Journal of Applied Physics*, 129(16):165103, Apr. 2021.

[5] D. Xu, S. Zhang, A. J. Liu, S. R. Nagel, and N. Xu. Discontinuous instabilities in disordered solids. *Proc. Natl. Acad. Sci. U.S.A.*, 120(34):e2304974120, Aug. 2023.
